# The STRING database in 2021: customizable protein–protein networks, and functional characterization of user-uploaded gene/measurement sets

**DOI:** 10.1093/nar/gkaa1074

**Published:** 2020-11-25

**Authors:** Damian Szklarczyk, Annika L Gable, Katerina C Nastou, David Lyon, Rebecca Kirsch, Sampo Pyysalo, Nadezhda T Doncheva, Marc Legeay, Tao Fang, Peer Bork, Lars J Jensen, Christian von Mering

**Affiliations:** Department of Molecular Life Sciences and Swiss Institute of Bioinformatics, University of Zurich, 8057 Zurich, Switzerland; Department of Molecular Life Sciences and Swiss Institute of Bioinformatics, University of Zurich, 8057 Zurich, Switzerland; Novo Nordisk Foundation Center for Protein Research, University of Copenhagen, 2200 Copenhagen N, Denmark; Department of Molecular Life Sciences and Swiss Institute of Bioinformatics, University of Zurich, 8057 Zurich, Switzerland; Novo Nordisk Foundation Center for Protein Research, University of Copenhagen, 2200 Copenhagen N, Denmark; TurkuNLP Group, Department of Future Technologies, University of Turku, 20014 Turun Yliopisto, Finland; Novo Nordisk Foundation Center for Protein Research, University of Copenhagen, 2200 Copenhagen N, Denmark; Novo Nordisk Foundation Center for Protein Research, University of Copenhagen, 2200 Copenhagen N, Denmark; Department of Molecular Life Sciences and Swiss Institute of Bioinformatics, University of Zurich, 8057 Zurich, Switzerland; Structural and Computational Biology Unit, European Molecular Biology Laboratory, 69117 Heidelberg, Germany; Molecular Medicine Partnership Unit, University of Heidelberg and European Molecular Biology Laboratory, 69117 Heidelberg, Germany; Max Delbrück Centre for Molecular Medicine, 13125 Berlin, Germany; Department of Bioinformatics, Biozentrum, University of Würzburg, 97074 Würzburg, Germany; Novo Nordisk Foundation Center for Protein Research, University of Copenhagen, 2200 Copenhagen N, Denmark; Department of Molecular Life Sciences and Swiss Institute of Bioinformatics, University of Zurich, 8057 Zurich, Switzerland

## Abstract

Cellular life depends on a complex web of functional associations between biomolecules. Among these associations, protein–protein interactions are particularly important due to their versatility, specificity and adaptability. The STRING database aims to integrate all known and predicted associations between proteins, including both physical interactions as well as functional associations. To achieve this, STRING collects and scores evidence from a number of sources: (i) automated text mining of the scientific literature, (ii) databases of interaction experiments and annotated complexes/pathways, (iii) computational interaction predictions from co-expression and from conserved genomic context and (iv) systematic transfers of interaction evidence from one organism to another. STRING aims for wide coverage; the upcoming version 11.5 of the resource will contain more than 14 000 organisms. In this update paper, we describe changes to the text-mining system, a new scoring-mode for physical interactions, as well as extensive user interface features for customizing, extending and sharing protein networks. In addition, we describe how to query STRING with genome-wide, experimental data, including the automated detection of enriched functionalities and potential biases in the user's query data. The STRING resource is available online, at https://string-db.org/.

## INTRODUCTION

Biomolecular networks are used pervasively in modern biology and medicine (1–3). They enable the inference of molecular functions through the ‘guilt-by-association’ principle (4,5), allow the characterization of modularity in biological processes (6–8) and serve as substrates for deep learning (9,10). They also support applications such as drug target discovery or drug repurposing (11,12), and can help in the interpretation of genomic variation (13). Biomolecular networks have been constructed for many different purposes and scopes, including networks of gene–gene regulatory events in transcription, networks of kinases/phosphatases and their substrates, or networks of metabolites together with the enzymes that interconvert them. One of the most useful, generic and broadly scoped network types is the protein–protein association network; it encompasses all protein-coding genes in a given genome, and highlights their functional associations (14). Since proteins can interact in many ways, a ‘functional association’ is typically defined operationally: any two proteins that jointly contribute toward a specific cellular process are deemed to be functionally associated (14–17); this definition even includes pairs of proteins that act antagonistically within the same process.

To construct a functional association network for the proteins of an organism, interaction evidence from a variety of sources needs to be considered; these sources may differ in their applicability depending on the proteins in question, their biological roles and the extent to which they have been studied experimentally. Data integration across different evidence sources is known to increase the overall network quality (18–21) and is also deemed necessary given the diverse modes by which proteins can be associated. The sources of interaction evidence fall into three broad classes: (i) prior knowledge—as available from curated pathway databases, or more generally from scientific publications, (ii) computational interaction predictions—from a variety of algorithms and (iii) direct lab experiments, using a variety of assays in both low- or high-throughput.

The STRING database is one of several online resources dedicated to organism-wide protein association networks. The field has been recently reviewed in (14,22). Frequently used resources include FunCoup (23), GeneMANIA (24), HumanBase/GIANT (25), IMP (26), IID (27), ConsensusPathDB (28) and HumanNet (29). These resources differ in terms of the types of interaction evidence they integrate, their organism coverage and the features of the web interfaces. STRING aims to place its focus on coverage (applying to thousands of genome-sequenced organisms), on completeness of evidence sources (e.g. including automated text mining) and on usability features (such as customization, enrichment detection and programmatic access). It allows users to log on and make their searches persistent, and it offers online-viewers to facilitate the inspection of the underlying evidence supporting each protein–protein association.

Apart from the website, the database can be queried directly from within Cytoscape (via a dedicated app) (30) and from within R (via a Bioconductor package) (31). STRING can also be queried programmatically for associations, network images or enrichments from any website or software, through its comprehensive REST API. All network items and scores, as well as all generated images and tables are freely available without restrictions, under the Creative Commons Attribution (CC BY 4.0) license. STRING has been selected as one of the *European Core Data Resources* by the ELIXIR consortium (32), is heavily cross-linked with other resources both within and outside of ELIXIR and is currently used by about 5000 distinct users per day.

## DATABASE CONTENT

The entire database content of STRING is pre-computed, stored in a relational database and available for separate download. All interaction evidence that contributes to a given network is benchmarked and scored (31,33–34), and the scores are integrated into a final ‘combined score’. This score is scaled between zero and one and provides an estimate of STRING’s confidence on whether a proposed association is biologically meaningful given all the contributing evidence. Each association is provided as a connection between two non-identical proteins, each from a different protein-coding gene locus. STRING does not differentiate between splicing variants or post-translationally modified protein isoforms encoded from the same locus—instead, all such isoforms are collapsed and represented by a single, canonical protein (i.e. a single protein per gene locus).

The various evidence types that contribute to STRING associations are first benchmarked and scored separately, in seven distinct *evidence channels*. These channels are also discernible in the visual STRING networks by lines of different colors, and they can be individually disabled by the user. The first three of the channels (*neighborhood*, *fusion* and *co-occurrence*) contain computational association predictions based on whole-genome comparisons. These so-called ‘genomic context’ channels can be computed for all organisms for which a completely sequenced genome is available, and do not depend on any further lab assays or measurements. In the case of the neighborhood channel, two proteins are given an association score when their encoding genes are in close proximity to each other on the chromosome. This channel is applicable mostly for Bacteria and Archaea; gene pairs achieve a higher score the closer they are on the chromosome (the distance is measured in terms of non-coding nucleotides between the two open reading frames). For the fusion channel, STRING scans all genomes for open reading frames that appear to be the result of gene-fusion events. For all inferred fusion events, the constituent, non-fused genes in other genomes/organisms are given an association score; the score is higher the better the fusion event can be delineated in terms of the orthology of the participating genes. The last of the genome context channels is the co-occurrence channel. Here, STRING searches for pairs of genes whose occurrence patterns throughout evolution show similarities. Such similarities can arise when genes have been transferred, lost, or duplicated together during evolution, which in turn can signify a shared function. For implementation details of this channel, refer to (37).

The next two channels are dealing with functional genomics experiments or direct lab assays. For the first (*co-expression*), STRING is collecting gene expression evidence from a number of sources; this is then normalized, pruned, and the expression profiles over a large variety of conditions are compared. Pairs of genes that show consistent similarities between their expression profiles are assigned association scores; the majority of the expression data is RNA-based, but we also import proteome expression data, from the ProteomeHD database (38). The *experiments* channel collects protein–protein interaction evidence from experiments and assays in the lab. This includes biochemical, biophysical and genetic experiments; all such interaction evidence is imported from the curated interaction database organized in the iMEX consortium (39), plus BioGRID (40).

The final two evidence channels deal with prior, consolidated knowledge on protein–protein associations. First, the *knowledge* channel parses association evidence from curated pathway databases, where it has been collected and consolidated manually by expert curators. These include pathways annotated in KEGG (41), Reactome (42) and MetaCyc (43), as well as protein complexes defined at the EBI Complex Portal (44) or by the Gene Ontology Consortium (45). Finally, statistical co-occurrence analysis across the scientific literature is performed for the *text-mining* channel. As of version 11.5 of STRING, the text-mining channel is based on PubMed abstracts (last updated on 28 July 2020), articles from the PMC open access subset (last updated on 17 April 2020) and text from OMIM (46) and SGD (47) entry descriptions. Pairs of proteins mentioned together in the same sentence, the same paragraph, or merely the same publication are assigned a benchmarked association score, the calculation of which is described in detail in (33).

## CUSTOMIZATION AND SHARING

The networks and reports generated by the STRING resource, whether for functional associations or physical interactions, can be configured and controlled through a number of options. First, users can broadly determine which types of evidence should be considered for a given network. All interaction evidence in STRING is thematically grouped into ‘channels’ (such as text mining, co-expression, lab experiments); these can be individually disabled by the user. Second, users can control the minimum score threshold below which interactions will not be shown. Third, enriched pathways of interest can be highlighted by coloring the respective proteins in the network, individually or in combinations. Fourth, the visual appearance of the network can be controlled, including whether or not singletons (i.e. unconnected proteins) are to be included, or what type of information should be highlighted through the styling of network edges. Lastly, the most versatile way of customizing a network is by adding additional custom data onto it, via the so-called ‘payload’ mechanism (see below). Together, these mechanisms allow highly specific networks and datasets to be generated. These customized networks can then be exported in a number of formats, including tabulated, machine-readable or visual formats. The same file formats and exports are also available for computational retrieval via a REST-based API interface. In addition, on most STRING views, a stable web-URL can be generated on request, which can be shared with other users; this URL is version-controlled and should not expire within the lifetime of a given STRING version.

**Figure 1.**
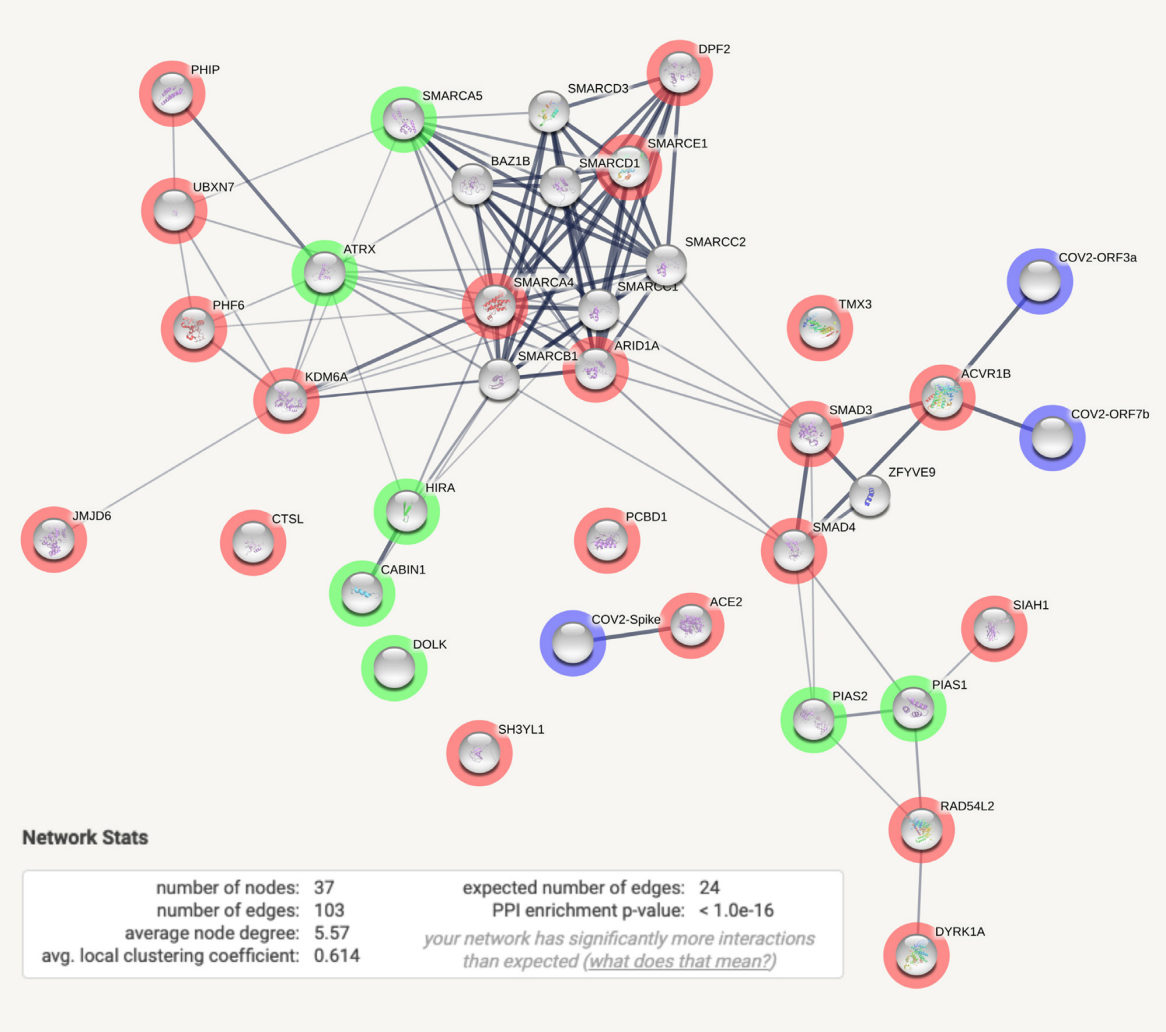
Example of a user-extended STRING network, adding external information. SARS-CoV-2 proteins, highlighted in blue, have been added to the standard human protein–protein association network in STRING, using the data add-on (‘payload’) mechanism. Virus proteins will automatically appear in the network based on their known associations with host proteins (as imported from the IMEx coronavirus interactome (35)). In addition, host proteins whose expression appears to control SARS-CoV-2 virion entry into cells, as determined in a recent genome-wide CRISPR-screen (36), are highlighted: proteins whose removal causes a drop in virus entry efficiency are highlighted in red; green highlights indicate proteins whose removal enhances virus entry. Proteins without highlights have entered the network based on close associations to the CRISPR screen proteins. The inset describes topological statistics of the network: it is strongly enriched in terms of functional associations, as compared to a random network of similar size.

For recurring users that have identified themselves through STRING’s login mechanism, additional options for generating and sharing content are available. Such users can generate and store their own gene lists; these can then be repeatedly re-visited and exported under a variety of settings. More importantly, they can upload and control self-provided add-on data (‘payload data’, see Figure 1). Such add-on data can be either node-centric, edge-centric or both. Through a node-centric payload, users can control several aspects of the appearance of a protein node, and can thereby communicate study-specific measurements or statistics of their own, for each node. Node color, textual annotation and links to external web resources can be customized for each node; in addition, a small space within the protein ‘popup’-window can be reserved for arbitrary HTML code. Similarly, the edge-centric settings include the possibility to customize the information that is shown for each protein–protein association, including textual annotations and extra markup. In addition, users can raise the confidence score of a given association (or create a novel association) based on additional evidence that they may have. This can be done either by raising the confidence score of one of STRING’s evidence channels, or by assigning a score to a dedicated ‘external’ channel reserved for this purpose. In either way, such user-assigned association scores contribute to the final combined score of an association and are fully searchable and browsable as part of the organism-wide network.

Together with user-provided payload data, an arbitrary legend image can be uploaded that explains and highlights the additions made, as well as a small banner image that is shown at the top of the page to alert the viewer to the added payload. In combination with the sharing mechanisms, this allows complex functional genomics datasets to be shared with other scientists, in an intuitive, searchable and browsable network context.

## ENRICHMENT DETECTION

An increasing number of STRING users enter the database not with a single protein as their query, but with a set of proteins. In this case, STRING will perform identifier mapping on the user's input and then display a network covering all the mapped proteins and their interconnections. As with all STRING networks, this can then be browsed interactively, inspected for the underlying evidence and clustered using k-means or MCL clustering. In addition, STRING will perform automated pathway-enrichment analysis on the user's input and list any pathways or functional subsystems that are observed more frequently than expected (using hypergeometric testing, against a statistical background of either the entire genome or a user-supplied background gene list). STRING will perform these overrepresentation tests for a total of eleven functional pathway classification frameworks, two of which are not available elsewhere. The commonly available frameworks are: Gene Ontology annotations (all three GO domains) (45), KEGG pathways (41), UniProt keywords (48), Reactome pathways (42), Pfam (49) and SMART (50) protein domains and InterPro protein features (51). Unique to STRING are the two remaining classification systems: i) a comprehensive name-tagged collection of the biomedical literature (PubMed abstracts, augmented by 2.7 million full-text articles), and ii) a hierarchical clustering of the STRING network itself, partitioned into smaller, tightly linked clusters. These two subsystems provide complementary and more exploratory enrichment views, compared to the established, manually annotated pathway classifications. In case of the publication-based system, individual publications assume the role of a pathway in enrichment testing: all proteins discussed in a given publication (identified using STRING’s text-mining pipeline) form a gene set, which is tested for over-representation on the user's input. With more than 3 million publications available for testing, this requires strong correction for multiple testing (52), but has the advantage of covering newly reported or controversial protein groupings that may yet have to appear in pathway databases. Likewise, the hierarchical STRING clustering provides protein groupings that are the result of a synthesis across all the interaction knowledge in the database, clustered to varying levels of stringency and hence to varying levels of functional granularity. These STRING clusters usually do not correspond fully to canonical pathways; they can include additional, less well-studied proteins and they may partition functional subsystems differently, which may or may not be better suited for any given user input. Apart from testing the STRING clusters separately, the website also reports a final test metric using the whole network: for each input gene set it is checked whether there are more interactions between the input proteins than expected for an input of that size.

For users entering with more than 2000 proteins at once, the network view becomes unwieldy; in addition, with such large inputs it can become relevant to know how the proteins are actually ranked *within* the input. For these cases, a new analysis mode is available since version 11.0 of STRING—it deals with large-scale input wherein each protein or gene comes with a user-provided numerical value. This allows the application of rank-based enrichment detection algorithms (functional class scoring). The user-provided value can be any relevant measurement or statistic, such as a log fold change, measured phenotype, mutation count, or expression strength. On such inputs, STRING will test the proteins of each known pathway for any non-random skew within the user-provided input values, and report statistically significant pathways. Of note, such functional class scoring approaches do not require a statistical background to be specified—the tests are applied solely within the user's input, and the rest of the genome is not considered. For this reason, users should provide the full list of available protein/value pairs as input, ideally genome-wide. STRING will test pathways for a skewed distribution on either end of the user's ranked input. Uniquely, STRING will also report pathways that are simultaneously enriched on *both* ends of the user's input (and thus depleted in the middle ranks). Kolmogorov–Smirnov testing is used to detect significant pathways, followed by Aggregate Fold-Change testing (53) where computationally feasible. The testing typically completes in <5 min, and the enriched pathways can be inspected and browsed interactively. Across the 11 gene annotation subsystems tested, STRING provides a comprehensive view of functional enrichments within a user's input, frequently including significant hits that are not reported elsewhere (for an instructive example, see ref (36)).

## CHANGES IN VERSION 11.5

Version 11.5 of STRING updates the organism coverage to 14 094, and includes a full re-import and re-scoring of all evidence types. In the experiments channel, the scoring has been revised to take into account the type of the assay that was used to detect the interaction in the lab. This information is now increasingly available, and STRING uses a globally benchmarked estimate of the relative performance of each assay type. In the text-mining channel, changes have been applied to allow the introduction of a document-specific stop-word list (‘stop-words’ are words that correspond to known gene names but appear too frequently and too unspecifically in texts to be used for entity recognition). This significantly increases the precision of the text-mining system. Specifically, we have created a high-confidence dataset consisting of millions of protein and non-protein text spans, which three widely adopted protein name recognition systems (54–56) agreed on tagging as such. Textual contexts of 200 words surrounding these spans have been used to create a high-confidence labeled dataset of positive and negative examples, which was subsequently used to train a deep learning-based model, taking outset in BioBERT (57), a state-of-the-art context-based biomedical language representation model. The deep learning model we trained can detect whether a text span is a protein, based on the context surrounding it. We used this model to generate the probability of being a protein for all matches of protein names, detected by the STRING text-mining system, in the scientific literature. Probabilities for the same names were combined within and across documents to automatically produce a list of the most problematic names detected by the text-mining system. Manual inspection of the list has assured its quality and it has been introduced to the text-mining pipeline, thus doubling the size of the previous manually curated stop-word list. In addition, a stop-word list that allows the resolution of ambiguous names at a document-specific level has been generated, using the same model. This list blocks names in ∼250000 specific documents, and unblocks valid protein names in ∼22000 documents, although these names are present in the stop-word list.

In the web interface, an important change is that users can now control the semantic meaning of edges in the network. The meaning (and scoring framework) can be set either to the traditional ‘functional association’, or limited to the ‘physical interaction’ subset; see below for more details.

**Figure 2.**
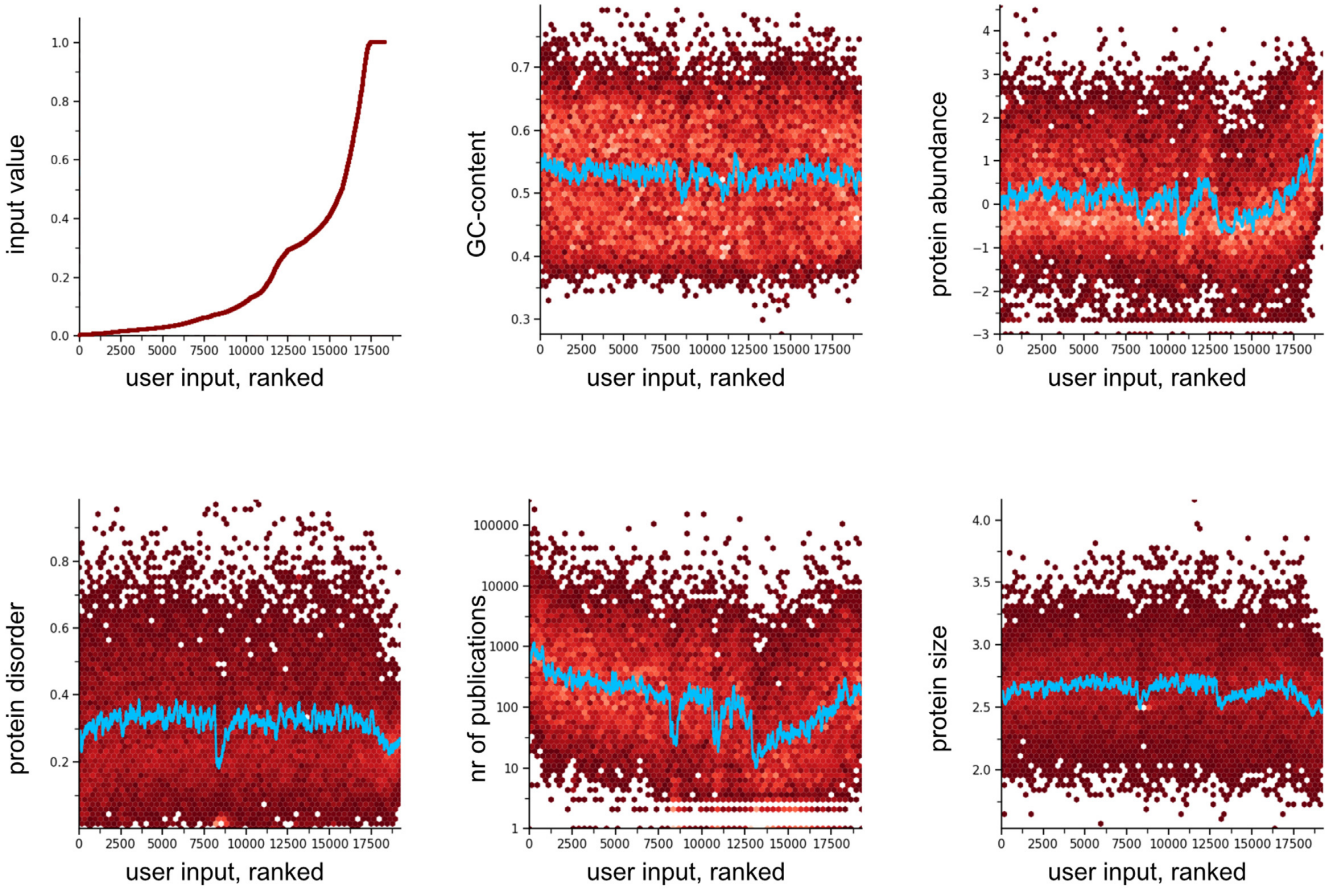
Example of a STRING-report on quantitative trends in a user input. Genome-scale inputs into STRING can be used to search for functional enrichments, but confounders in the data can potentially complicate interpretation. A new STRING feature allows to visualize such confounding trends. Here, STRING was queried with a large set of human proteins, whereby each protein was entered together with its approximate likelihood of being targeted toward the mitochondrion (‘Mito Evidence IMPI score’, from the MitoMiner database (59)). As expected, to rank proteins by their mitochondrial localization likelihood reveals no trend in terms of the GC content of their encoding genes, but noticeable trends in some of the other measures tested. Protein abundance is taken from PaxDB (60), expressed in parts-per-million (log-scale). The ‘nr of publications’ refers to the tagged corpus of the STRING text-mining channel, counting how many publications have been tagged for a given protein with at least one of its known names. The protein size corresponds to the amino-acid length of the canonical isoform expressed at a given gene locus (log-scale).

Another change concerns the functional enrichment analysis, specifically in the case of large inputs with experimental measurements associated to each protein/gene. Functional enrichment analysis in such genome-scale experiments can be influenced by inherent biases—which can be either technical or biological in origin (58). Beginning in version 11.5 of STRING, therefore, an automated bias analysis is performed for large-scale user inputs. This occurs in the background, while the enrichment testing is executed, and results in a graphical report showing potential systematic biases/trends in the input (Figure 2). Currently, the potential confounders that are tested include i) average protein abundance, ii) protein length, iii) number of publications mentioning the gene or protein in PubMed-indexed literature, iv) protein disorder as predicted by IUPred and v) average GC content of the encoding transcript.

## PHYSICAL INTERACTIONS MODE

While protein–protein associations in STRING by definition are functional, i.e. not necessarily physical interactions, the organization of proteins in a physical complex provides particularly strong evidence for their biological relationship. Therefore, in addition to the functional channel and combined scores, we now also assign physical interaction scores to associations in STRING, if the proteins show evidence of co-occurring in a complex. Physical interaction scores are calculated for selected evidence channels and aggregated into a combined physical interaction score, which can be specifically selected by the user at query time.

To derive the physical interaction score, a gold standard dataset of trusted protein complexes is required. As opposed to functional relationships, such datasets are available only for a few organisms. Therefore, the scores derived from a benchmark of physical interactions in one of these organisms have to be applied to all other organisms contained in STRING. We chose *Saccharomyces cerevisiae* as our gold standard organism due to the extensive experimental work carried out over the past decades to identify protein interactions in this model organism. This has led to a comprehensive set of well-established interactions being distilled in various databases. Among these, the Complex Portal (44) database provides a sufficient number of stringently manually curated yeast protein complexes, covering a broad range of functional areas, to be used as our gold standard dataset for physical interactions.

To score the physical interactions identified by evidence from the experiments channel, all interactions derived only from genetic interference methods are excluded as purely functional and the remaining interactions are benchmarked against the gold standard of protein complexes, similarly to the functional association benchmark (34). For physical interactions however, a protein pair is considered a true positive if both proteins are found together in any gold standard complex, which means they can be directly or indirectly interacting. True and false positive interactions were down-weighted during benchmark by the geometric mean of the gold standard node degrees to account for the large number of protein pairs in large complexes. The physical interaction score is then the probability of two proteins being together in a gold standard complex. A calibration function derived from relating the physical with the functional channel scores is used to assign a physical interaction score to all physical interactions that are not covered by the gold standard dataset. Finally, we apply the yeast calibration curve to the experiments channel data of other species to derive their physical channel scores from their functional channel scores.

For the text-mining channel, a dedicated pipeline had to be developed to extract physical interaction information from the literature, since the fact that proteins are mentioned together in text is not enough to infer that they also physically interact. BioBERT (57) served once again as the basis for the development of a deep learning-based relation extraction text-mining model to extract physically interacting protein pairs from the scientific literature. The model we developed has been trained on an unbiased dataset of 6145 manually annotated relations, extracted both from PubMed abstracts and PMC full-text articles, and can predict whether two proteins are in a complex together, based on the context around them. We used this model to generate a physical interaction probability for all sentences in the scientific literature mentioning protein pairs, and then combined these probabilities first within and then across documents to generate a raw physical interaction score for each unique pair of proteins using the following scoring function:

$$\begin{equation*}\left( {1 - p} \right) = \left( {1 - {p^*}} \right)\ \prod \frac{{\left( {1 - {p_i}} \right)}}{{\left( {1 - {p^*}} \right)}}\end{equation*}$$

where *p_i_* is the probability of physical interaction for protein pair *i* within a document, *p* is the probability of physical interaction for the same pair across documents and p* is the prior probability of a sentence mentioning a pair of physically interacting proteins.

Similar to the benchmarking of interactions for the experiments channel, the text-mined yeast interactions are ranked by their raw score and benchmarked against the gold standard of protein complexes to calculate the final physical interaction scores for the text-mining channel. As an additional control, we also benchmarked the human text-mined interactions against the human protein complexes in Complex Portal. The fact that the resulting relationship between the raw text-mining and physical interaction scores is similar for the two distant organisms further supports our decision to use one calibration curve for all organisms.

## CONCLUSION

Taken together, the network and enrichment facilities in STRING enable comprehensive characterization of user gene lists and functional genomics datasets, and allow the creation and sharing of highly customized and augmented protein–protein association networks. Future work in STRING will include options to prune the networks down to specific cell-types or tissues based on gene expression information, as well as further expansions of the functional enrichment detection to additional classification systems and more complex types of user input.
