# Correction to ‘The STRING database in 2021: customizable protein–protein networks, and functional characterization of user-uploaded gene/measurement sets’

**DOI:** 10.1093/nar/gkab835

**Published:** 2021-09-16

**Authors:** Damian Szklarczyk, Annika L Gable, Katerina C Nastou, David Lyon, Rebecca Kirsch, Sampo Pyysalo, Nadezhda T Doncheva, Marc Legeay, Tao Fang, Peer Bork, Lars J Jensen, Christian von Mering

**Affiliations:** Department of Molecular Life Sciences and Swiss Institute of Bioinformatics, University of Zurich, 8057 Zurich, Switzerland; Department of Molecular Life Sciences and Swiss Institute of Bioinformatics, University of Zurich, 8057 Zurich, Switzerland; Novo Nordisk Foundation Center for Protein Research, University of Copenhagen, 2200 Copenhagen N, Denmark; Department of Molecular Life Sciences and Swiss Institute of Bioinformatics, University of Zurich, 8057 Zurich, Switzerland; Novo Nordisk Foundation Center for Protein Research, University of Copenhagen, 2200 Copenhagen N, Denmark; TurkuNLP Group, Department of Future Technologies, University of Turku, 20014 Turun Yliopisto, Finland; Novo Nordisk Foundation Center for Protein Research, University of Copenhagen, 2200 Copenhagen N, Denmark; Novo Nordisk Foundation Center for Protein Research, University of Copenhagen, 2200 Copenhagen N, Denmark; Department of Molecular Life Sciences and Swiss Institute of Bioinformatics, University of Zurich, 8057 Zurich, Switzerland; Structural and Computational Biology Unit, European Molecular Biology Laboratory, 69117 Heidelberg, Germany; Molecular Medicine Partnership Unit, University of Heidelberg and European Molecular Biology Laboratory, 69117 Heidelberg, Germany; Max Delbrück Centre for Molecular Medicine, 13125 Berlin, Germany; Department of Bioinformatics, Biozentrum, University of Würzburg, 97074 Würzburg, Germany; Novo Nordisk Foundation Center for Protein Research, University of Copenhagen, 2200 Copenhagen N, Denmark; Department of Molecular Life Sciences and Swiss Institute of Bioinformatics, University of Zurich, 8057 Zurich, Switzerland

*Nucleic Acids Research*, 2021, 49(Database): D605–D612, https://doi.org/10.1093/nar/gkaa1074

When this paper first published, it only had one corresponding author, Christian von Mering. It should have had three corresponding authors: Christian von Mering, Peer Bork, and Lars J. Jensen. This has now been corrected online.

